# Unsustainable anthropogenic mortality disrupts natal dispersal and promotes inbreeding in leopards

**DOI:** 10.1002/ece3.6089

**Published:** 2020-03-18

**Authors:** Vincent N. Naude, Guy A. Balme, Justin O'Riain, Luke T.B. Hunter, Julien Fattebert, Tristan Dickerson, Jacqueline M. Bishop

**Affiliations:** ^1^ Institute for Communities and Wildlife in Africa University of Cape Town Cape Town South Africa; ^2^ Panthera New York NY USA; ^3^ Wildlife Conservation Society Bronx NY USA; ^4^ Centre for Functional Biodiversity School of Life Sciences University of KwaZulu‐Natal Durban South Africa; ^5^ Wyoming Cooperative Fish and Wildlife Research Unit Department of Zoology and Physiology University of Wyoming Laramie WY USA

**Keywords:** home‐range, kin‐clustering, microsatellites, *Panthera pardus*, philopatry, relatedness

## Abstract

Anthropogenic mortality of wildlife is typically inferred from measures of the absolute decline in population numbers. However, increasing evidence suggests that indirect demographic effects including changes to the age, sex, and social structure of populations, as well as the behavior of survivors, can profoundly impact population health and viability. Specifically, anthropogenic mortality of wildlife (especially when unsustainable) and fragmentation of the spatial distribution of individuals (home‐ranges) could disrupt natal dispersal mechanisms, with long‐term consequences to genetic structure, by compromising outbreeding behavior and gene flow. We investigate this threat in African leopards (*Panthera pardus pardus*), a polygynous felid with male‐biased natal dispersal. Using a combination of spatial (home‐range) and genetic (21 polymorphic microsatellites) data from 142 adult leopards, we contrast the structure of two South African populations with markedly different histories of anthropogenically linked mortality. Home‐range overlap, parentage assignment, and spatio‐genetic autocorrelation together show that historical exploitation of leopards in a recovering protected area has disrupted and reduced subadult male dispersal, thereby facilitating opportunistic male natal philopatry, with sons establishing territories closer to their mothers and sisters. The resultant kin‐clustering in males of this historically exploited population is comparable to that of females in a well‐protected reserve and has ultimately led to localized inbreeding. Our findings demonstrate novel evidence directly linking unsustainable anthropogenic mortality to inbreeding through disrupted dispersal in a large, solitary felid and expose the genetic consequences underlying this behavioral change. We therefore emphasize the importance of managing and mitigating the effects of unsustainable exploitation on local populations and increasing habitat fragmentation between contiguous protected areas by promoting *in situ* recovery and providing corridors of suitable habitat that maintain genetic connectivity.

## Graphical abstract

Unsustainable anthropogenic mortality of wildlife and fragmentation of the spatial distribution of individuals disrupts natal dispersal mechanisms, with long‐term consequences to genetic structure, by compromising outbreeding behavior and gene flow. Our study investigates this threat in African leopards, demonstrating novel evidence directly linking unsustainable anthropogenic mortality to inbreeding through disrupted dispersal in a large, solitary felid and exposes the genetic consequences underlying this behavioral change.

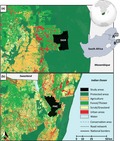

## 1 INTRODUCTION

When assessing the effects of anthropogenic mortality on wildlife populations, managers and policymakers typically consider only direct numerical responses of populations to human‐mediated mortality (poaching, retaliatory conflict, and unregulated trophy hunting; Woodroffe & Ginsberg, 1998). Indirect demographic effects (age, sex, and social structure) and the behavior of survivors have profound impacts on the health and viability of remaining populations (Ausband, Mitchell, Stansbury, Stenglein, & Waits, 2017; Ausband, Stansbury, Stenglein, Struthers, & Waits, 2015; Rutledge et al., 2010). For example, harvest can facilitate the spatial reorganization of individuals within populations by creating home‐range vacancies that can be filled by neighboring or immigrant conspecifics through a “vacuum effect” (Frank, Leclerc, et al., 2017a). This may increase the probability of encounters between unfamiliar individuals leading to elevated rates of conflict, sexually selected infanticide, and increased local extinction risk (Creel et al., 2015, 2016; Gosselin, Zedrosser, Swenson, & Pelletier, 2015; Whitman, Starfield, Quadling, & Packer, 2004). Moreover, directed harvest toward a specific sex, age, or size cohort disrupts dispersal patterns (Frank, Ordiz, et al., 2017b; Milner, Nilsen, & Andreassen, 2007).

By maintaining gene flow within and among populations, dispersal is critical to the persistence of spatially structured metapopulations (Dolrenry, Stenglein, Hazzah, Lutz, & Frank, 2014; Gundersen, Johannesen, Andreassen, & Ims, 2001; Hanski & Simberloff, 1997). However, by increasing territorial turnover and providing opportunities for subadults to settle locally, harvest limits natal dispersal (in the absence of immigration), affecting both local and metapopulation dynamics (Blyton, Banks, & Peakall, 2015; Newby et al., 2013). While many studies highlight the demographic effects of unsustainable harvest, the behavioral mechanisms employed to counteract these effects and subsequent consequences to population genetic structure remain poorly understood, particularly in large carnivores (Milner et al., 2007). While inbreeding susceptibility is documented in felids (e.g., *Panthera leo*, Munson et al., 1996; *Puma concolor*, Ernest, Vickers, Morrison, Buchalski, & Boyce, 2014), few monitoring studies have the requisite longitudinal mortality (well‐documented mortality for entire populations), spatial (fine‐scale movement of known individuals), and genetic (multigenerational pedigrees of known individuals) data to enable comparison between populations and thereby demonstrate a tenable link between high levels of mortality (often human‐mediated), disrupted dispersal, and inbreeding (Onorato, Desimone, White, & Waits, 2011).

Across southern Africa, large felids have a long history of both legal and illegal exploitation. African leopards (*Panthera pardus pardus*) have been heavily harvested throughout this region for their economic value as trophies in legal hunts (Balme, Slotow, & Hunter, 2010; Braczkowski et al., 2015; Swanepoel, Lindsey, Somers, Hoven, & Dalerum, 2011) and for mostly illegal use in traditional practices (Harries, 1993; Kumalo & Mujinga, 2017; Williams, Loveridge, Newton, & MacDonald, 2017). Many leopards are also removed in retaliatory conflict due to their real or perceived threat to livestock (Loveridge, Wang, Frank, & Seidensticker, 2010). In this study, we investigate how such anthropogenic mortality and persecution disrupt individual dispersal in leopards, altering spatial patterns of kinship, which ultimately promotes inbreeding in this solitary species. Previous telemetry studies suggest that leopards, like many polygynous mammals, generally exhibit female philopatry and male‐biased natal dispersal (Balme, Robinson, Pitman, & Hunter, 2017a; Fattebert, Balme, Dickerson, Slotow, & Hunter, 2015a; Fattebert et al., 2016). Subadult females are thus predicted to compete for philopatry and attempt to breed within or adjacent to their natal ranges, forming spatially defined kin‐clusters (Lambin, Aars, & Piertney, 2001). In contrast, subadult male leopards typically disperse in order to avoid competition with larger, conspecific adult males, thereby reducing the probability of mating with related females (Dobson, 1982; Wolff, 1994). In heavily harvested populations, young male leopards are released from local male–male competition and may exhibit “opportunistic natal philopatry” to avoid the substantial costs of dispersal, undertaking shorter dispersal distances and establishing home‐ranges nearer their mothers and sisters (Fattebert, Robinson, Balme, Slotow, & Hunter, 2015b). In such a scenario, the socio‐spatial structure of males is expected to approximate the kin‐clustered spatial structure of females, which, in the absence of active inbreeding avoidance, ultimately promotes increased levels of localized inbreeding (Støen, Bellemain, Sæbø, & Swenson, 2005).

Here, we use home‐range estimates together with parentage and relatedness analyses to explore dispersal dynamics and the consequent fine‐scale genetic structure of two leopard populations with markedly different histories of anthropogenically linked mortality: a well‐protected population at ecological carrying capacity (Balme, Pitman, et al., 2017b; Balme et al., 2019) and a population recovering from a recent history of extensive anthropogenic mortality (Balme, Slotow, & Hunter, 2009; Balme et al., 2010). Under the premise of density‐dependent male‐biased dispersal and female philopatry, we predict that (a) female leopards with overlapping home‐ranges will support higher levels of relatedness than males in both populations, this being particularly evident in the recovering population where mothers can adjust their home‐ranges to accommodate daughters—whereas this would not always be possible in a population at capacity (Fattebert et al., 2016); (b) levels of relatedness between overlapping males and females will be higher in the recovering population due to reduced dispersal distances of sons (Fattebert, Robinson, et al., 2015b); and (c) reduced dispersal distances exhibited by both sexes in the recovering population will result in higher levels of inbreeding. We discuss our findings in the context of local population fitness and the broader implications of disrupted dispersal on persistence and functional connectivity across leopard metapopulations throughout protected areas.

## 2 MATERIALS AND METHODS

### 2.1 Study areas

**Figure 1:**
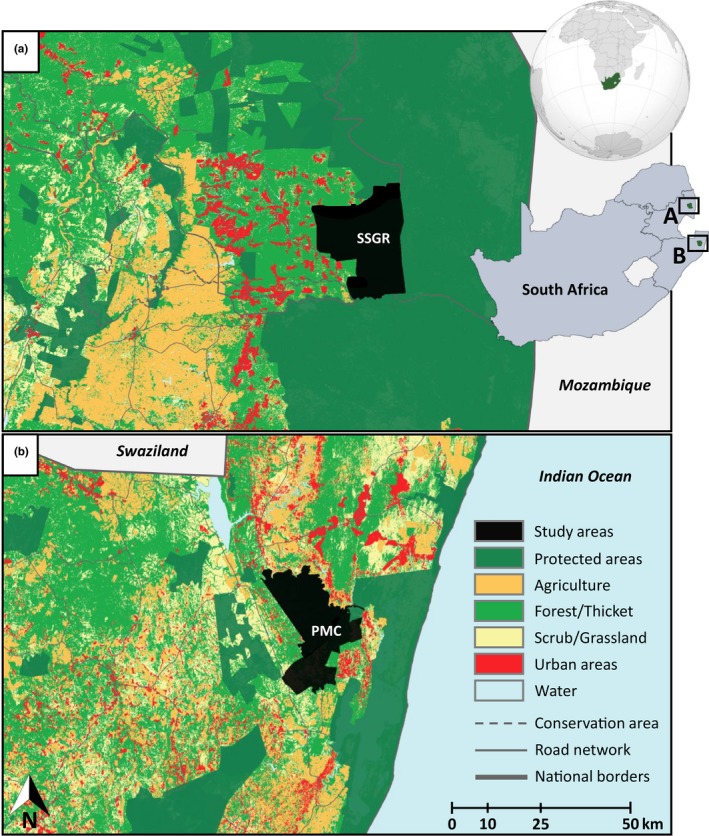
Maps showing the position of the two study areas within the existing matrix of land use and habitat type. SSGR: Sabi Sand Game Reserve (a) and PMC: Phinda‐uMkhuze Complex (b) indicated in black

The study was undertaken in two protected area complexes of South Africa that differ markedly in their historical rates of anthropogenic mortality. The Sabi Sand Game Reserve (SSGR) is a privately owned conservancy (est. 1948) in the Lowveld region of the Mpumalanga province (Figure 1a). It covers 625 km^2^ but is contiguous along its southern and eastern boundaries with the Kruger National Park and Manyeleti Game Reserve in the north. The SSGR thus forms part of a much larger (~22,000 km^2^) protected system. Although the western boundary of the reserve is adjacent to a densely populated community, the border fence is impermeable to leopards and the population seems unaffected by detrimental edge effects (Balme et al., 2019). There is also no legal offtake of leopards inside the SSGR and levels of poaching are very low; anthropogenic mortality accounted for <2% of leopard deaths in the SSGR between 1975 and 2015 and the population appears at capacity (Balme, Pitman, et al., 2017b).

The Phinda‐uMkhuze Complex (PMC) is situated in the Maputaland region of the KwaZulu‐Natal province (Figure 1b) and comprises two neighboring reserves: Phinda Private Game Reserve (est. 1991) and the public uMkhuze Game Reserve (est. 1912), forming a contiguous protected landscape of 660 km^2^. The PMC is surrounded by a mosaic of commercial game ranches, livestock farms, and Zulu communities; these land types are often hostile to leopards (Thorn, Green, Dalerum, Bateman, & Scott, 2012). Unlike the SSGR, the boundary fence of the PMC is permeable to leopards and individuals move freely between protected and unprotected land (Balme et al., 2010). The PMC, particularly uMkhuze, also suffers high levels of wire‐snare poaching, which can have a marked effect on large carnivores such as leopards (Becker et al., 2013). Accordingly, leopards in the PMC face far greater mortality risk than those in the SSGR; between 2002 and 2012, human‐related mortality accounted for >50% of all leopard deaths in the PMC (Balme et al., 2009). Nonetheless, recent policy changes have allowed the PMC leopard population to recover: from a disturbance period (pre‐2004), when the population was in decline (λ = 0.978); through a recovery period (2005–2008), following the implementation of sustainable harvest protocols and other conservation interventions (λ = 1.136); to a stabilization period (2009–2012), when the population density reached putative carrying capacity (λ = 1.010; Fattebert, Robinson, et al., 2015b).

Historically, the SSGR and PMC populations were possibly linked via dispersal (Fattebert, Hunter, Balme, Dickerson, & Slotow, 2013). The two study sites also have similar habitats (open to semi‐wooded savannah), climates (mean monthly temperatures ranging from 19 to 33°C and average annual rainfall of ~600 mm), levels of prey abundance, and similar leopard densities (SSGR: 11.81 ± 2.56 leopards 100/km^2^, Balme et al., 2019; PMC: 9.51 ± 1.22 leopards 100/km^2^ following recovery, Rogan et al., 2019), forming contiguous leopard habitat with no physical barriers to dispersal (Figure S1). Accordingly, the observed differences in spatial behavior and genetic structure are assumed to be the result of human interference rather than due to other environmental or ecological factors, such as competitor presence or density which does not differ between these reserves (Balme, Pitman, et al., 2017b; Balme et al., 2019; Fattebert et al., 2016; Rogan et al., 2019).

### 2.2 Data collection and sampling

In the SSGR, individual location data were collected through direct observation of leopards, using methods detailed in Balme, Pitman, et al. (2017b). Briefly, the SSGR hosts several ecotourism lodges that operate high‐end photographic safaris. Clients are taken on “game‐drives” twice daily led by an experienced guide and tracker. The high density of vehicles (98 ± 2 per game drive) and extensive road network (mean road density of 3.2 km per km^2^) ensures that most of the reserve is traversed daily at a high expected vehicle encounter rate (0.17 ± 0.05 vehicles per km). Drives are not limited to roads, as skilled trackers pursue charismatic species by vehicle or on foot until the animal is located, or the tracks are lost. This intensive search effort results in frequent sightings; on average, 6,428 ± 914 unique leopard sightings are recorded per annum with individual leopards being seen on average every 2.74 ± 0.04 days. Leopards in the SSGR are highly habituated to vehicles and guides are familiar with the individuals residing in their traversing area (individual leopards can be distinguished by their unique vibrissae patterning; Miththapala, Seidensticker, Phillips, Fernando, & Smallwood, 1989). Data captured include the identity of the individual leopard (if known), GPS location of the sighting, the presence and number of offspring, as well as other notable behavior (e.g., intra‐ and interspecific interactions). Although multiple guides sometimes submitted data from the same sighting, we retrospectively filtered the data to ensure that each unique sighting was captured only once, that is, an individual leopard was included in only a single sighting per game drive. To assess the accuracy of the guides' ability to distinguish individuals, we asked them to submit photographs with the putative identity of the animal from a random subset of sightings; they correctly identified the individual leopard (*n* = 121) in all photographs. We also cross‐referenced data submitted by guides from different lodges to assess the consistency of the information captured, and we found no significant discrepancies (as in Balme et al., 2013). Samples for DNA analysis were obtained from leopard fecal deposits collected by guides in the SSGR. Only samples where the guide observed the leopard defecating (and they were therefore confident of its identity) were used in analyses. In total, 145 samples from 81 individuals were collected between 2015 and 2018. Fecal samples were dry‐stored on silica beads at −80°C.

Spatial data in the PMC were collected using telemetry, following methods detailed in Fattebert et al. (2016). Leopards were captured using a combination of free‐darting, cage‐trapping, and soft‐hold foot‐snaring and fitted with either a VHF (250 g, Sirtrack Ltd., Havelock North, New Zealand, 0.5% of adult female body mass) or GPS (420 g, Vectronic‐Aerospace, Berlin, Germany, 1.2% of adult female body mass) collar. VHF‐collared individuals (*n* = 41) were located every three days on average to within ~100 m using ground homing or triangulation across the PMC (mean road density of 2.6 km per km^2^), whereas GPS collars (*n* = 28) were programmed to record 2–6 fixes daily. Ear‐punch biopsy samples from 69 individuals were collected for genetic analyses during captures from 2002 to 2012. Tissue samples were stored in >90% ethanol at −20°C. Capture and collaring of leopards were approved (research permit HO/4004/07) by the provincial conservation authority, Ezemvelo KwaZulu‐Natal Wildlife and by the Animal Ethics Subcommittee of the University of KwaZulu‐Natal Ethics Committee (approval 051/12/Animal).

### 2.3 Home‐range estimation

To determine the spatial distribution and dispersal patterns of individuals in the two study populations, we calculated home‐range estimates (size, centroid, utilization density, and overlap) for all sexually mature (≥3 years) leopards postdispersal (Balme et al., 2013), using autocorrelated kernel density estimates (ADKEs; Fleming et al., 2015), where an ANOVA was used to identify significant differences in relocation counts between individuals of different spatial sampling types (observation, GPS, and VHF). These 95% AKDEs are considered robust for comparisons between different spatial data types (Fleming et al., 2015). All pairwise comparisons of spatial overlap between individuals were restricted to periods of temporal co‐occurrence (over a continuous four‐year sampling period of three generations). Subsequent analyses were focussed on home‐range overlap (HRO; Bhattacharyya coefficient), as the most relevant metric with regard to inbreeding opportunity, as this relates directly to encounter potential and is not affected by between‐site variance in home‐range size (km^2^). Variogram calculations, movement model fits, and home‐range estimations were implemented in the *ctmm* package (Calabrese, Fleming, & Gurarie, 2016; Fleming et al., 2015). Home‐range centroids were estimated as the geometric mean of coordinates used to fit the AKDE contours.

Estimated semi‐variance was plotted as a function of time‐lag to visually inspect the autocorrelative structure of the location data (Fleming et al., 2014). Brownian motion (BM) or Ornstein–Uhlenbeck (OU) movement models were used at zero to short time lags, where a linear increase in the semi‐variance corresponded with uncorrelated velocity, whereas integrated OU (IOU) or OU with foraging (OUF) was used where upward curvature at these time lags indicated autocorrelation in the velocity. If plotted semi‐variance did not approach an asymptote, individuals were not considered to be range residents; these leopards were either not monitored for long enough or did not exhibit behaviors that meet the definition of range residents and were removed from further analyses. Thereafter, space use was investigated by assessing behavior across longer time lags, where range residents are expected to reach an asymptote on a timescale that corresponds to the home‐range crossing time (Calabrese et al., 2016; Fleming et al., 2014). Maximum‐likelihood model fits (Fleming et al., 2014) were ranked by AICc (Calabrese et al., 2016). Home‐ranges were estimated conditionally on the fitted and selected model per individual. OU models are described using two parameters (home‐range crossing time in days and variance in km^2^), while OUF models are described using three (home‐range crossing time in days, velocity autocorrelation timescale in hours, and variance in km^2^). OU models provided home‐range and crossing time estimates, where OUF models provided these metrics as well as the velocity autocorrelation timescale and average distance travelled per individual. Finally, volumetric space‐time UD and HRO (Bhattacharyya's coefficient) were estimated based on these selected models (Fieberg & Kochanny, 2005; Winner et al., 2018). All analyses were conducted in *R* (R Core Team, 2018) and *QGIS* (QGIS Development Team, 2018).

### 2.4 DNA extraction, PCR, and genotyping

DNA was successfully extracted for 81 individuals from SSGR and 69 individuals from PMC. DNA was extracted from fecal samples using the QIAamp DNA Stool Mini Kit and from tissue using the DNEasy Blood and Tissue Kit (Qiagen, Inc., Valencia, CA, USA). Individuals were genotyped at 22 microsatellite loci (Table S1) previously shown to be polymorphic in leopards (McManus et al., 2014; Ropiquet et al., 2015; Uphyrkina et al., 2001) together with a Zn‐finger linked sexing marker (Pilgrim, McKelvey, Riddle, & Schwartz, 2004). PCRs contained ~50–100 ng/µl DNA, 200 ng/μl bovine albumin serum (BSA), a locus‐specific MgCl_2_ concentration (1.5–2.5mm), 2.0 μm each of forward‐labeled and reverse primers, 5 μl DreamTaq^™^ Green PCR Master Mix (Thermoscientific), and deionized water to a total reaction volume of 25 μl. PCRs were performed on an Applied Biosystems Veriti^®^ Thermal Cycler. Given the generally lower quality DNA extracted from fecal samples, all samples were amplified in singleplex and in triplicate (from extraction to amplification) to ensure reproducibility. Locus‐specific thermal profiles were developed following Menotti‐Raymond et al. (1999), and PCR products were pooled according to size and fluorescent labeling for visualization (Table S1). A positive control was used for size scoring between runs, and a negative control was included throughout. Genotypes were analyzed on a 3100‐Avant Genetic Analyzer (Applied Biosystems) at the Central Analytical Facility, Stellenbosch University, South Africa. Genotypes were sized using the LIZ^®^ 600 internal size standard and alleles were scored in GENEIOUS R10 (Biomatters Limited). Automated allele calls were manually checked for accuracy. Genotyping error was assessed per triplicate sample run on each individual and ≥2/3 consensus alleles used in subsequent analyses, where no such consensus was achieved or genotypes failed (≤15/22 loci amplified), whole genotypes were removed. Where available, known parent–offspring relationships were used to find mismatches. Stutter errors, large allele dropouts, short allele dominance, and significant departures from Hardy–Weinberg equilibrium (HWE) were examined across loci for each population using a chi‐square test for goodness of fit and sequential Bonferroni corrections performed on the resulting *P*‐values (Rice, 1989). FSTAT 2.9 was used to test for linkage disequilibrium (LD) between pairs of loci (Goudet, 2002). The significance of sex ratio estimates for each population was assessed with a binomial distribution test, calculated as the probability of the observed number of males and females given an expected sex ratio of 0.5.

### 2.5 Kinship, relatedness, and inbreeding

Parentage assignment and relatedness indices were used to confirm kinship and augment our observed pedigrees for both populations. Individual parentage assignments were estimated within a maximum‐likelihood framework implemented in CERVUS 3.0 (Kalinowski, Taper, & Marshall, 2007). Simulations were generated at a given level of confidence for all offspring analyzed. Parameters included the following: 100,000 offspring, 2% mistyped loci, 89% typed loci for SSGR, and 93% typed loci for PMC, as determined by CERVUS for the dataset. Assignment was only tested if a minimum of 15 loci were successfully genotyped, while candidate parents were limited to adults (≥3 years old) and pairs that were alive at the same time. Parents were assigned based on likelihood‐of‐difference (LOD) scores calculated at both 95% (strict) and 80% (relaxed) confidence levels. The strict assignment (95%) was used to build whole pedigrees, whereas the more relaxed assignment (80%) was used to provide further insight into likely relationships between individuals when not strictly assigned. Where no 95% assignment was supported and a clear 80% assignment was available, this was used to assign parentage. Pairwise relatedness between all individuals in both populations was estimated using the Wang relatedness metric (*r*_w_) in SPAGeDI 1.0 (Hardy & Vekemans, 2002; Wang, 2002). This estimator was chosen for its apparent desirable properties among reviewed relatedness indices, namely, low sensitivity to the sampling error that results from estimating population allele frequencies and a low sampling variance that decreases asymptotically to the theoretical minimum with increasing numbers of loci and alleles per locus (Blouin, 2003). For each population, the frequency distribution of relatedness coefficients was summarized for defined kin‐categories (unknown, parent–offspring, full‐sibling, half‐sibling, and breeding pairs) based on field observations (e.g., mothers with offspring, siblings), parentage analysis, and relatedness scores. Observed pedigrees were supported and expanded for both populations and inbreeding events recorded. In addition, the *adegenet* (Jombart, 2008) and *ape* (Paradis & Schliep, 2018) R‐packages were used to estimate per locus and population‐level inbreeding coefficients (*F_IS_*).

### 2.6 Spatio‐genetic structure

To test for evidence of restricted or disrupted dispersal, we examined the fine‐scale genetic structure of offspring (mother–daughter [M‐D]; mother–son [M‐S]) and sex‐based dyads (female–female [F‐F]; female–male [F‐M]; male–male [M‐M]) per unit distance from the natal range in our two study populations. We first superimposed assigned maternal home‐range centroids with a concentric ring (the average maternal home‐range area) surrounded by three concentric rings representing: the nearest‐neighboring maternal home‐range (1st order); the next peripheral neighboring maternal home‐range (2nd order); and all other maternal home‐range areas beyond this periphery. The width of each band represents the average maternal home‐range radius by population. Offspring (those assigned through parentage analyses) home‐range centroids were then plotted in relative x‐y proximity to their natal centroid and their frequencies plotted by concentric ring so as to schematically represent the differences in philopatric home‐range establishment relative to the natal home‐range by sex for each population.

We then quantified the association between matrices of pairwise genetic and spatial distances (Peakall, Ruibal, & Lindenmayer, 2007; Smouse & Peakall, 2001) through direct correlation, spatial autocorrelation analysis and mantel tests implemented in the *ecodist* package (Goslee & Urban, 2007). Under a restricted or disrupted dispersal model, autocorrelograms yield positive correlations at short spatial distances (classes represent the average home‐range diameter per sex and population), followed by a gradual decrease to zero with increasing geographical distance and a subsequent random fluctuation of positive and negative values of the correlation coefficient (Smouse & Peakall, 2001). The first x‐intercept estimates the extent of nonrandom genetic structure or defines the point at which random stochastic drift replaces gene flow as the key determinant of genetic structure (Vangestel, Mergeay, Dawson, Vandomme, & Lens, 2011). As this intercept is dependent upon the true scale of genetic structure, the chosen distance class size, and the sample size per distance class (Peakall et al., 2007), we also performed a second autocorrelation analysis in which we plotted pairwise genetic distances against increasing inclusive distance classes. Here, the distance class at which the autocorrelation coefficient no longer remains significant (999 bootstraps) approximates the true extent of identifiable genetic structure between groups of individuals (Peakall et al., 2007).

## 3 RESULTS

### 3.1 Home‐range estimates

Home‐ranges (km^2^) were successfully estimated for all 142 adult leopards for which we had genetic data (SSGR: females = 49; males = 24; total = 73; PMC: females = 31; males = 38; total = 69). Due to high sampling intensity, rare forays or peripheral movements were witnessed (mostly among young males) and accounted for in all three datasets, where home‐range relocation counts did not differ significantly between individuals of different spatial sampling types ( ${\bar{x}}_{\mathrm{observed}}$ = 336 ± 7.20 [SE]; ${\bar{x}}_{\mathrm{GPS}}$ = 367 ± 14.20 [SE]; ${\bar{x}}_{\mathrm{VHF}}$ = 361 ± 11.7 [SE]; *F*_2_ = 2.99; *p* = .05). Male home‐ranges were markedly larger than that of females in both the SSGR ( ${\bar{x}}_{\mathrm{female}}$ = 26.93 ± 2.37 [SE]; ${\bar{x}}_{\mathrm{male}}$ = 50.02 ± 5.43 [SE]; *t*_32_ = 3.90; *p* < .001) and PMC ( ${\bar{x}}_{\mathrm{female}}$ = 31.54 ± 1.34 [SE]; ${\bar{x}}_{\mathrm{male}}$ = 50.32 ± 5.01 [SE]; *t*_44_ = 3.57; *p* < .001). Female and male home‐range size did not differ between study populations (Tables S1 and S3).

### 3.2 Genotyping and genetic diversity

The final dataset (Table S4) consisted of 15–21 loci successfully typed for 142 known individuals in our two study populations. “Extraction to genotyping success” (number of repeats required per sample) was significantly lower in the PMC than in the SSGR ( ${\bar{x}}_{\mathrm{PMC}}$ = 1.04 ± 0.03 [SE]; ${\bar{x}}_{\mathrm{SSGR}}$ = 2.56 ± 0.09 [SE]; *t*_81_ = 15.69; *p* < .001; CI = −1.71, −1.33) and genotyping failed (<15/22 loci amplified successfully) for only eight leopards (SSGR = 8; PMC = 0). Locus FCA096 was removed from all further analyses due to poor amplification success (14%–30% of individuals). There was no evidence of LD or scoring errors due to large allele dropout and stutter in either population. Mean genotype coverage was higher in SSGR than in PMC ( ${\bar{x}}_{\mathrm{SSGR}}$ = 68.05 ± 0.39 [SE]; ${\bar{x}}_{\mathrm{PMC}}$ = 63.52 ± 1.57 [SE]; *t*_40_ = 2.80; *p* = .008; CI = 1.26, 7.79). SSGR supports greater heterozygosity ( ${\bar{x}}_{\mathrm{SSGR}}$ = 0.78 ± 0.03 [SE]; ${\bar{x}}_{\mathrm{PMC}}$ = 0.65 ± 0.03 [SE]; *t*_40_ = 3.01; *p* < .005; CI = 0.04, 0.22), allelic richness ( ${\bar{x}}_{\mathrm{SSGR}}$ = 6.01 ± 0.31 [SE]; ${\bar{x}}_{\mathrm{PMC}}$ = 4.89 ± 0.26 [SE]; *t*_38_ = 2.78; *p* = .008; CI = 0.30, 1.94), and mean number of private alleles per locus than PMC ( ${\bar{x}}_{\mathrm{SSGR}}$ = 2.86 ± 0.37 [SE]; ${\bar{x}}_{\mathrm{PMC}}$ = 0.52 ± 0.13 [SE]; *t*_24_ = 5.90; *p* < .001; CI = 1.52, 3.15). With the exception of some locus‐level deviations, the SSGR population was in HWE (see Table S4), whereas PMC was not, with 12 out of the 21 markers out of HWE. The SSGR showed significant female bias (z = 2.81, *p* = .003), with no significant sex bias in PMC (z = −0.72, *p* = .235). As there are likely very few unknown individuals in both populations, these sex ratios are assumed to reflect absolute sex ratios and are thus not expected to create a bias in overlap measures.

### 3.3 Parentage analysis, relatedness, and inbreeding

Formal computational assignment of parentage, via a likelihood framework, was successful for both populations (Table S5). Maternity (no paternity known) was assigned for 63% of offspring in SSGR and for 48% of offspring in PMC, corroborating all 30 putative field‐based maternal assignments in SSGR and 25/31 maternal assignments in PMC. Paternity (given known maternity) was assigned for a 64% of offspring in PMC and 54% in SSGR, confirming all 20 putative sires in SSGR and 16/28 in PMC. Biparental assignment was not possible for 30% of offspring in SSGR and 19% in PMC, while the predicted resolving power (95% confidence) of loci sampled was higher in SSGR (99%) than in PMC (96%), with 1% and 4% assigned with 80% confidence in SSGR and PMC, respectively.

**Figure 2:**
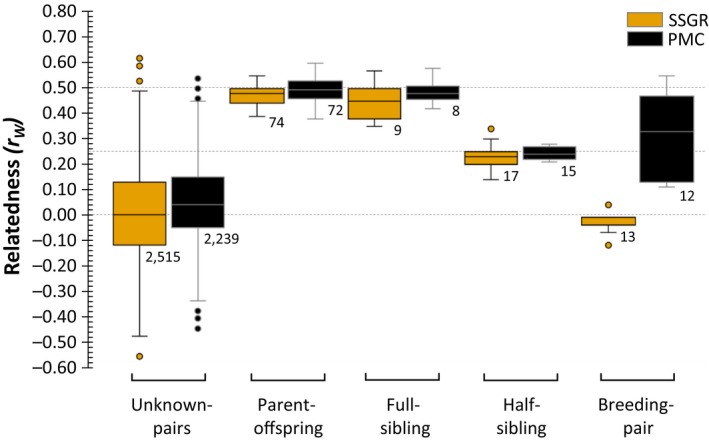
Pairwise relatedness estimates (r_w_) of confirmed kinship categories. Expected theoretical relatedness coefficients for parent–offspring/full siblings (0.5), half‐sibling (0.25), and unrelated/random pairs (0) are indicated by dashed lines. The distribution for each kinship category and number of pairs (below boxes) is indicated for Sabi Sand Game Reserve (gold) and the Phinda‐uMkhuze Complex (black)

In both populations, kinship pairs showed mean relatedness coefficients within the limits of their expected distributions (Figure 2), including confirmed breeding pairs in SSGR which were significantly less related than random ( $\bar{x}$ = −0.05; *t*_12_ = 4.27; *p* = .001; CI = −0.08, −0.03). Mean relatedness of confirmed breeding pairs in PMC, however, did not fall within the limits of their expected distribution ( $\bar{x}$ = 0.31; *t*_11_ = 6.07; *p* < .001; CI = 0.19, 0.42). Instead, these were more similar to that expected of the half‐sibling distribution ( $\bar{x}$ = 0.31; *t*_11_ = 1.09; *p* = .297; CI = −0.06, 0.17).

Pedigree reconstruction provided no evidence of direct inbreeding in SSGR, whereas in PMC, one father–daughter and two half‐sibling mating events were identified. The inbreeding coefficient (*F*_IS_) was significantly greater in PMC than in SSGR (Table S4; ${\bar{x}}_{\mathrm{SSGR}}$ = −0.08 ± 0.02 [SE]; ${\bar{x}}_{\mathrm{PMC}}$ = 0.06 ± 0.03 [SE]; *t*_40_ = 4.93; *p* < .001; CI = −0.20, 0.08), with evidence of significant outbreeding in SSGR with *F*_IS_ scores significantly less than 0 ( ${\bar{x}}_{\mathrm{SSGR}}$ = −0.08 ± 0.02 [SE]; *t*_20_ = 5.27; *p* < .0001; CI = −0.11, −0.05) and significant levels of inbreeding in PMC with *F*_IS_ scores significantly greater than 0 ( ${\bar{x}}_{\mathrm{PMC}}$ = 0.06 ± 0.03 [SE]; *t*_20_ = 2.58; *p* < .05; CI = 0.01, 0.12).

### 3.4 Spatio‐genetic structure

The mean proportion of home‐range overlap (Table 1) among all individuals was higher in PMC than SSGR ( ${\bar{x}}_{\mathrm{SSGR}}$ = 0.16 ± 0.00 [SE]; ${\bar{x}}_{\mathrm{PMC}}$ = 0.20 ± 0.00 [SE]; *t*_2088_ = 2.90; *p* < .001). While the proportion of home‐range overlap was not significant between populations for female–female and male–male dyads, female–male home‐range overlap was significantly higher in PMC than in SSGR ( ${\bar{x}}_{\mathrm{SSGR}}$ = 0.15 ± 0.01 [SE]; ${\bar{x}}_{\mathrm{PMC}}$ = 0.20 ± 0.01 [SE]; *t*_1036_ = 3.22; *p* = .001). Home‐range overlap between kin‐related pairs was not significant, with the exception of mother–son pairs in PMC being twice that of SSGR ( ${\bar{x}}_{\mathrm{SSGR}}$ = 0.31 ± 0.10 [SE]; ${\bar{x}}_{\mathrm{PMC}}$ = 0.61 ± 0.06 [SE]; *t*_9_ = 2.49; *p* = .034) and breeding pair home‐range overlap being nearly 20% greater in SSGR than PMC ( ${\bar{x}}_{\mathrm{SSGR}}$ = 0.63 ± 0.05 [SE]; ${\bar{x}}_{\mathrm{PMC}}$ = 0.45 ± 0.07 [SE]; *t*_18_ = 2.11; *p* = .049). Home‐range overlap between mother–daughter pairs was slightly greater than mother–son pairs in SSGR ( ${\bar{x}}_{\mathrm{FD}}$ = 0.55 ± 0.06 [SE]; ${\bar{x}}_{\mathrm{MS}}$ = 0.31 ± 0.10 [SE]; *t*_9_ = 2.03; *p* = .073).

**Table 1:** Pairwise home‐range overlap of 95% autocorrelated kernel density estimates (AKDE), described as the utilization density (Bhattacharyya coefficient) per dyad, confirmed kin‐relationships, breeding pairs, and across all individuals for 142 known leopards within the Sabi Sand Game Reserve (SSGR) and Phinda‐uMkhuze Complex (PMC), South Africa, 2002–2018. Parameter estimates are presented as the percentage of population pairs with overlap (%); mean proportion of home‐range utilization overlap ( $\bar{x}$ ); standard errors (SE); and associated P‐values are based on the *t*‐statistic for independent variables (two‐tailed), with Welch correction for unequal variance, where confidence intervals are presented (CI)

Category	SSGR		PMC		Comparison		
	%	$\bar{x}$ (±SE)	%	$\bar{x}$ (±SE)	*t*_df_	*p*‐value	CI
All Individuals	45.51	0.16 (0.00)	43.22	0.20 (0.00)	4.54_2088_	<.001***	−0.07; 0.03
Dyads							
Female–Female	48.13	0.15 (0.00)	44.52	0.18 (0.02)	1.48_334_	.140	−0.07; 0.00
Female–Male	44.47	0.15 (0.01)	44.14	0.20 (0.01)	3.22_1036_	.001**	−0.08; −0.02
Male–Male	38.79	0.18 (0.02)	40.83	0.22 (0.01)	1.27_200_	.203	−0.09; 0.02
Kin‐relationships							
Father–Daughter	63.16	0.49 (0.08)	76.47	0.34 (0.06)	1.61_18_	.1241	−0.04; 0.33
Father–Son	33.33	0.32 (0.14)	39.13	0.39 (0.11)	0.41_6_	.694	−0.51; 0.36
Mother–Daughter	70.37	0.55 (0.06)	92.86	0.59 (0.07)	0.44_27_	.666	−0.24; 0.15
Mother–Son	48.73	0.31 (0.10)	81.15	0.61 (0.06)	2.49_9_	.034*	−0.55; −0.05
Breeding Pairs	100	0.63 (0.05)	83.33	0.45 (0.07)	2.11_18_	.049*	0.00; 0.37
SSGR							
Father–Daughter/Father–Son			(*N* = 19/12)		1.07_4_	.344	−0.27; 0.61
Mother–Daughter/Mother–Son			(*N* = 27/16)		2.03_9_	.073˙	−0.03; 0.52
PMC							
Father–Daughter/Father–Son			(*N* = 17/23)		0.39_10_	.706	−0.31; 0.22
Mother–Daughter/Mother–Son			(*N* = 14/18)		0.14_25_	.893	−0.21; 0.18

^˙^ *p* ≤ 0.10; * *p* ≤ 0.05; ** *p* ≤ 0.01; *** *p* ≤ 0.001.

**Figure 3:**
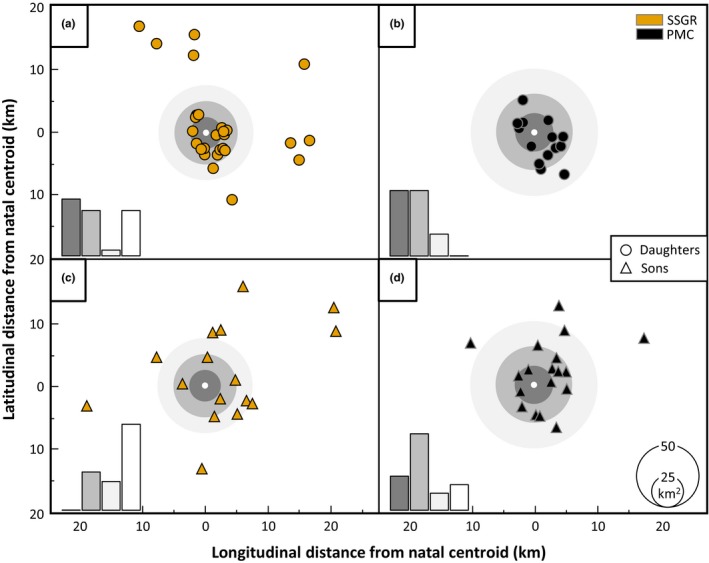
Spatial distribution of postdispersal offspring relative to their natal home‐range. Postdispersal centroids for daughters (circles) and sons (triangles) are shown relative to their superimposed maternal centroids (white circles) for SSGR (gold) and PMC (black). Rings of gray indicate the area of successional average female home‐range (95% ADKE) radii around the natal centroid; three levels are shown: the maternal home‐range (dark gray), the 1^st^‐order peripheral home‐range (gray), and the 2^nd^‐order peripheral home‐range. A linear summary of the proportion of individuals in each category is provided (bar graph bottom left)

Of the 27 daughters (Figure 3a) and 16 sons (Figure 3c) assigned in SSGR, 37% of daughters and no sons established home‐range centroids within their mean maternal home‐ranges, 30% of daughters and 25% of sons within the 1^st^ order mean peripheral home‐range, 3% of daughters and 19% of sons within the 2^nd^ order, and 30% of daughters and 56% of sons beyond. In contrast, of the 14 daughters (Figure 3b) and 18 sons (Figure 3d) assigned in PMC, 43% of daughters and 22% of sons established home‐range centroids within their mean maternal home‐ranges, 43% of daughters and 50% of sons within the 1^st^ order mean peripheral home‐range, 14% of daughters and 11% of sons within the 2^nd^ order, and no daughters and 17% of sons beyond.

**Figure 4:**
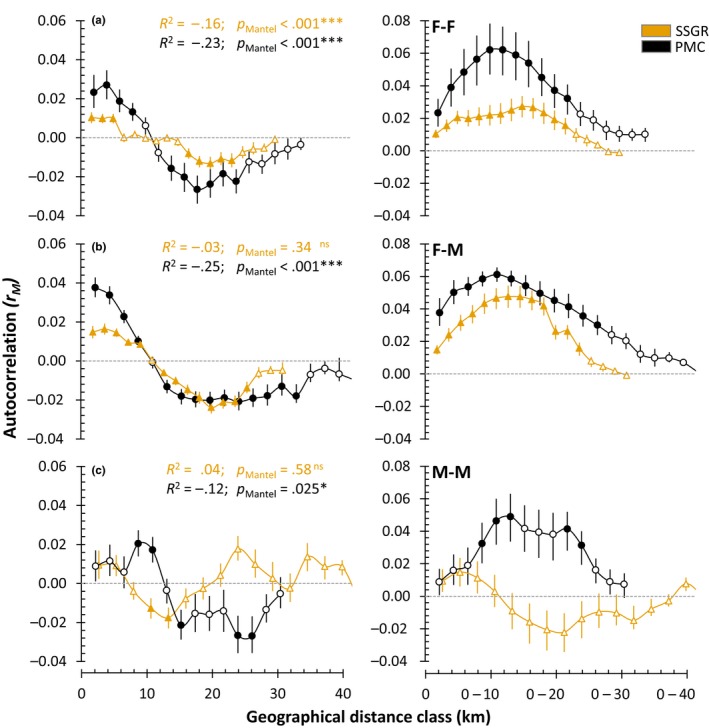
Spatial autocorrelation of pairwise relatedness estimates (rw) over geographical distance (km) are indicated for the Sabi Sands Game Reserve (gold triangles) and the Phinda‐uMkhuze Complex (black circles) by female–female (a), female–male (b), and male–male (c) dyads, respectively. Depicted as a function of geographical distance (left) and as the effect of different distance class sizes on the extent of genetic autocorrelation (right). Significant spatio‐genetic autocorrelation is indicated by solid shapes and its direction determined above or below the dashed 0‐line. Hollow shapes indicate nonsignificance or an independent spatio‐genetic pattern within the distance class

Mantel tests showed population‐level spatio‐genetic structuring in both populations (Figure 4). Pairwise relatedness (*r*_w_) decreased significantly as the proximity (km) between individuals increased within female–female dyad pairs in SSGR (*R* ^2^ = −.16; *p* < .001) and within female–female (*R* ^2^ = −.23; *p* < .001), female–male (*R* ^2^ = −.25; *p* < .001), and male–male (*R* ^2^ = −.12; *p* = .025) dyad pairs in PMC (Figure 4; Table S3). Autocorrelograms revealed fine‐scale spatio‐genetic structure by pairwise proximity between individuals in each dyad. Female kin‐clustering was observed in both populations (Figure 4a), where significantly positive autocorrelation occurred over three female home‐range radii in SSGR (0–6 km) and four in PMC (0–8 km). This female kin‐clustering effect was stronger over these distances in the PMC. Significant clustering of females that were less related than expected at random occurred for a range beyond this distance in both populations (SSGR = 16–24 km; PMC = 14–24 km), while all spatio‐genetic structure showed no significant autocorrelation (spatio‐genetic independence) beyond 24 km in both reserves. While this relationship is stronger in PMC than SSGR, significantly positive female–male kin‐clustering occurred over five home‐range radii in SSGR and four in PMC (Figure 4b). Significant clustering of unrelated individuals then occurred for a range beyond this distance in both populations (SSGR = 12–26 km; PMC = 12–34 km), while all spatio‐genetic structure attenuated beyond 24–28 km in both reserves. The spatio‐genetic structure of male–male dyad pairs was largely independent throughout both populations (Figure 4c), with some isolated incidents of significant autocorrelation. Spatio‐genetic structure in SSGR was independent, while bimodal structure occurred in PMC (8–14; 21–25 km) which attenuated beyond 26 km.

## 4 DISCUSSION

In this study, we contrast leopard populations from SSGR (a well‐protected population at carrying capacity) and PMC (a postharvest population in recovery) to reveal the fine‐scale genetic consequences of disrupted dispersal due to these markedly different histories of anthropogenic mortality. As predicted, mothers shared >50% of their home‐ranges with their daughters in both populations. A consequence of this female philopatry is the spatial formation of adult female kin‐clusters, a phenomenon evidenced by the strong spatio‐genetic autocorrelation in female–female dyads in both SSGR and PMC. Matrilineal assemblages are typical among large, solitary carnivores, having been observed in brown bears (*Ursus arctos*; Støen et al., 2005), pumas (Sweanor, Logan, & Hornocker, 2001), and tigers (*Panthera tigris*; Smith, 1993; Goodrich et al., 2010; Gour et al., 2013). Strategies to deal with the costs of increased resource competition (for food and mates) implicit in this conservative dispersal by females are assumed to have evolved because of the increased inclusive fitness benefits that accrue—the so‐called “resident fitness hypothesis” (Anderson, 1989; Lambin et al., 2001). This is clearly evident in the recovering PMC, where daughters do not establish beyond the 2^nd^‐order mean peripheral home‐range of mothers. Here, historical anthropogenic mortality may have created “gaps” in the spatial matrix allowing mothers to accommodate daughters within their home‐ranges (Balme, Robinson, et al., 2017a). Female kin‐clustering and natal philopatry are evident in SSGR; however, unexpectedly, 30% of daughters appear to have dispersed beyond their maternal home‐ranges. As this population is considered to be at capacity (Balme, Pitman, et al., 2017b), this may be novel evidence of density‐dependent female dispersal, as postulated by Fattebert, Robinson, et al. (2015b).

Subadult males disperse from their natal range at sexual maturity (~3 years old) to avoid conflict with resident adult males (Fattebert et al., 2016; Fattebert, Robinson, et al., 2015b) and inbreeding with related females (Balme et al., 2019). While kin‐recognition mechanisms have evolved in many species to limit close inbreeding, sex‐biased natal dispersal is the primary outbreeding mechanism of most polygynous mammals and is essential to maintaining gene flow within and among populations (Greenwood, 1980). Sexually mature male leopards in SSGR conformed to this inbreeding avoidance/mate competition paradigm with no sons establishing within their maternal home‐ranges. However, in PMC 22% of sons established home‐ranges overlapping with their maternal home‐range, suggesting a disruption in the proximate drivers of male dispersal, leading to reduced dispersal and opportunistic philopatry, in congruence with Fattebert, Robinson, et al. (2015b). This is further supported by a strong negative correlation between relatedness and distance in female–male dyads in PMC, resulting in population‐level male kin‐clustering similar to that of females. While this phenomenon of disrupted dispersal has been reported in large carnivores with cooperative breeding strategies (Loveridge, Searle, Murindagomo, & Macdonald, 2007), it has rarely been documented in solitary species (Riley et al., 2014), and to our knowledge, this is the first evidence of population‐level male kin‐clustering in a large solitary felid.

Male kin‐clustering in polygynous mammals increases the likelihood of opportunistic mating events with close female relatives (sisters, mothers, aunts, and cousins) which, without kin‐recognition (Støen et al., 2005), may result in local inbreeding (Matocq & Lacey, 2004; Perrin & Mazalov, 2000). In our study, mean relatedness scores among confirmed breeding pairs in SSGR were essentially random (Figure 2), with low population‐level *F*_IS_ scores indicative of significant outbreeding. This result was expected, as there is likely outbreeding and effective gene flow throughout the contiguous Kruger National Park landscape. The high degree of relatedness (half‐sibling) among breeding pairs in PMC however suggests that historically high levels of anthropogenic mortality promote opportunistic male philopatry and kin‐clustering with translates into significant population‐level inbreeding (high *F*_IS_ scores). While behavioral avoidance alone does not seem to be a strong enough driver of dispersal, as local inbreeding was observed (father–daughter and half‐sibling) in PMC, it may be muting even stronger population‐level inbreeding signals. Similar findings of reduced dispersal and outbreeding benefits linked to sustained harvest have been documented in pumas (Logan & Sweanor, 2000; Sweanor, Logan, & Hornocker, 2001), bobcats (*Lynx rufus*; Johnson, Walker, & Hudson, 2010), and black bears (*Ursus americanus*; Moore, Draheim, Etter, Winterstein, & Scribner, 2014). While the PMC leopard population is currently recovering from high levels of anthropogenically‐linked mortality (Rogan et al., 2019), demographic‐based metrics alone do not reveal the loss of genetic diversity and the consequences this may have for the future health and viability of the population (Kendall et al., 2009). Our results thus further highlight the importance of population connectivity to ensure gene flow and genetic diversity through immigration (Fattebert, Robinson, et al., 2015b; Frankham, 2003; Hauenstein et al., 2019).

Potential alternative explanations for this observed pattern include within‐reserve habitat fragmentation and/or typical density‐dependent dispersal contributing to the differences between these populations, because they are at different stages of “development.” The former posits that high levels of human‐caused mortality (such as in the PMC) are correlated with anthropogenic barriers to movement (Tucker et al., 2018); however, there is no evidence to suggest that anthropogenic barriers limit leopard dispersal in either of these populations (Figure 1), and if this were the primary force behind limited dispersal, it would still not explain why these barriers are sex‐specific (Figure 4). The latter suggests that the SSGR has been stable for some time, while the PMC is recovering and has only recently stabilized, such that increasing density to parity with SSGR (+2.3 leopards per km^2^) might correct male dispersal and ultimately outbreeding. Though demographic recovery is plausible, this does not mitigate the “genetic scaring” (evident in reduced heterozygosity and increased inbreeding) accrued by the PMC population and many small reserves like it, when undergoing fluctuations of extreme harvest. Without immigration and effective connectivity between these reserves (an increasingly scarce alternative), genetic recovery through drift alone may not be rapid enough, as the ongoing genetic resilience of these populations is compromised and at risk of stochastic effects. Moreover, mortality need not be unsustainable to induce these effects, as it is not known whether “sustainable mortality” by humans would necessarily eliminate the patterns observed. Certainly, less mortality would have a mitigating effect, but it is not known to what degree. Our study is limited by the comparison of only two reserves on the wide spectrum of anthropogenic mortality and its impacts. Unfortunately, the fine‐scale genetic structure of African leopard populations remains understudied. This hinders the use of heterozygosity, relatedness scores, and conventional inbreeding coefficients as a means of interpreting population‐level effects, as there is no baseline data on allelic frequency and diversity of “natural” (outbred or panmictic) populations. Despite this, we are encouraged that multiple lines of evidence derived from both spatial and genetic data provide consistent support for anthropogenic mortality driving limited dispersal in males which in turn results in kin‐clustering and ultimately inbreeding.

Given time and adequate protection, territorial turnover among male leopards in PMC may slow and stabilize, re‐establishing male‐biased dispersal and restoring the typical in situ outbreeding effect of genetic drift as capacity is reached (Couvet, 2002; Fattebert, Robinson, et al., 2015b). An alternative is leopard translocation under a metapopulation management approach; however, translocations have been largely unsuccessful to date, as leopards are wide‐ranging, have complex social dynamics, and are costly to contain (Athreya, Odden, Linnell, & Karanth, 2010; Ropiquet et al., 2015; Weilenmann, Gusset, Mills, Gabanapelo, & Schiess‐Meier, 2010; Weise, Stratford, & Vuuren, 2014). Genetic recovery and ultimate sustainability could more likely be managed and fast‐tracked by formally maintaining connectivity between PMC and its surrounding reserves (e.g., Makhasa Nature Reserve, Ubombo Mountain Nature Reserve, Isimangaliso Wetland Park, Manyoni Private Game Reserve, Thanda Safari—Big 5 Game Reserve and Hluhluwe‐iMfolozi Park). Wildlife corridors have proven successful in maintaining functional gene flow between populations in large, solitary felids like jaguar (Wultsch et al., 2016) and tiger (Sharma et al., 2013), despite the political (e.g., land ownership, conservation priorities) and logistical (e.g., road networks, suitable habitat) challenges.

Few protected areas sufficiently encompass the wide range of these species and large, solitary carnivores effectively confined to small reserves often suffer edge effects and even localized extinction (Woodroffe & Ginsberg, 1998). Our study demonstrates novel genetic consequences underlying this process and emphasizes the importance of managing and mitigating the effects of increasingly threatened protected areas and fragmented corridors of structurally suitable habitat that maintain effective connectivity (Fattebert, Balme, et al., 2015a; Hauenstein et al., 2019; Kaiser, 2001).

## CONFLICT OF INTEREST

None declared.

## AUTHORS' CONTRIBUTIONS

VN, GB, and JB conceived the study and designed the experimental protocols with input from JOR. Data were collected by VN, GB, LH, JF, and TD. Analyses were conducted by VN and JB. All authors discussed and interpreted the data. VN, GB, LH, JF, TD, JOR, and JB contributed critically to the writing of the manuscript and gave final approval for publication.

## Data Availability

Summaries of calculated metrics used in analyses are reported in the appendix, and any further information can be made available upon request from the corresponding author.
