# Supplementary material for: Unsustainable anthropogenic mortality disrupts natal dispersal and promotes inbreeding in leopards
Source: Ecol Evol. 2020 Mar 18;10(8):3605–19. doi: 10.1002/ece3.6089 (PMC7160178; doi:10.1002/ece3.6089)
Supplement: Supplementary file 1 [file ECE3-10-3605-s001.docx]

**
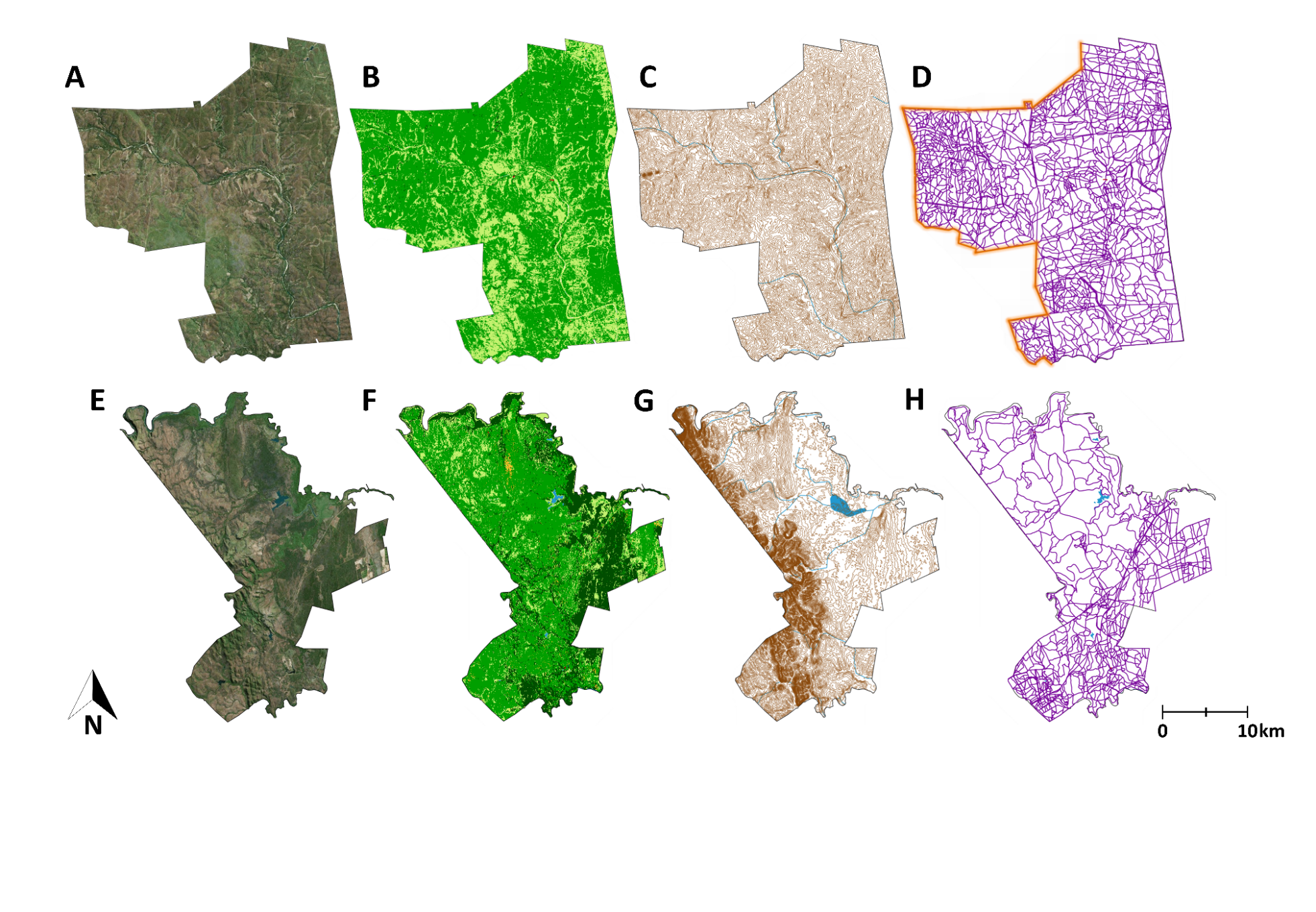
**

**Figure A1.** Iterative maps of the Sabi Sands Game Reserve (A–D) and Phinda-uMkhuze Complex (E–H). Indicated are (left to right): high-resolution aerial imagery; landcover (this layer key is identical to that of Figure 2); relative elevation (5m contours) as well as the two-track or graded road networks (purple) and leopard impermeable fences (orange) in both reserves. These serve to show that leopard habitat is maintained and contiguous throughout both reserves and indeed beyond these borders with no evidence of limited connectivity through physical barriers to dispersal and subsequent genetic connectivity.

**Table A1.** Microsatellite markers used to genotype 142 known leopards at 22 loci and a sex-linked marker in the Sabi Sand Game Reserve (SSGR) and Phinda-uMkhuze Complex (PMC), South Africa, 2002–2018. Described by post single reaction multiplex pool, forward and reverse primer sequence, product size (bp), florescent tag used and single reaction-specific annealing temperature. Panels were developed in order to maximize comparability with existing studies (Menotti-Raymond et al., 1999; Uphyrkina et al., 2001; McManus et al., 2014; Ropiquet et al., 2015 and Pilgrim et al., 2005).

| **Multiplex pool** | **µSat locus** | **Forward primer sequence (5’–3’)** | **Reverse primer sequence (5’–3’)** | **Product size (bp)** | **Florescent tag** | **Annealing temp. (°C)** |
| --- | --- | --- | --- | --- | --- | --- |
| 1 | Zn-Finger | AAGTTTACACAACCACCTGG | CACAGAATTTACACTTGTGCA | 163, 166 | FAM | 48 |
| 1 | FCA008 | ACTGTAAATTTCTGAGCTGGCC | TGACAGACTGTTCTGGGTATGG | 122 – 148 | VIC | 56 |
| 2 | FCA026 | GGAGCCCTTAGAGTCATGCA | TGTACACGCACCAAAAACAA | 136 – 154 | NED | 56 |
| 2 | FCA043 | GAGCCACCCTAGCACATATACC | AGACGGGATTGCATGAAAAG | 116 – 128 | FAM | 56 |
| 3 | FCA075 | ATGCTAATCAGTGGCATTTGG | GAACAAAAATTCCAGACGTGC | 103 – 143 | FAM | 54 |
| 2 | FCA077 | GGCACCTATAACTACCAGTGTGA | ATCTCTGGGGAAATAAATTTTGG | 143 – 155 | FAM | 56 |
| 2 | FCA090 | ATCAAAAGTCTTGAAGAGCATGG | TGTTAGCTCATGTTCATGTGTCC | 93 – 120 | VIC | 56 |
| 3 | FCA094 | TCAAGCCCCATTTTACCTTC | CACCTGAGCCAAAGGCTATC | 215 – 237 | FAM | 56 |
| 2 | FCA096 | CACGCCAAACTCTATGCTGA | CAATGTGCCGTCCAAGAAC | 184 – 224 | VIC | 54 |
| 1 | FCA097 | TAATGTTCAACTTGAATTGCTTCC | GAACAGTAGTTTGCCCATACAGG | 138 – 148 | NED | 56 |
| 3 | FCA105 | TTGACCCTCATACCTTCTTTGG | TGGGAGAATAAATTTGCAAAGC | 189 – 197 | PET | 52 |
| 1 | FCA126 | GCCCCTGATACCCTGAATG | CTATCCTTGCTGGCTGAAGG | 139 – 145 | FAM | 56 |
| 3 | FCA139 | AGCATGTTTTTGAGGCAGCT | TTATGGGTAGTGTGAAGTAGGG | 141 – 147 | NED | 56 |
| 1 | FCA161 | TTACCGATACACACCTGCCA | CACAGACGTGCTCTAGCCAA | 179 – 187 | FAM | 56 |
| 1 | FCA211 | TGTAGAACATAATGCCTCAGCC | TCTTGAACCTATTTCCCCACA | 111 – 119 | PET | 52 |
| 1 | FCA220 | CGATGGAAATTGTATCCATGG | GAATGAAGGCAGTCACAAACTG | 214 – 222 | VIC | 56 |
| 1 | FCA224 | CTGGGTGCTGACAGCATAGA | TGCCAGAGTTGTATGAAAGGG | 154 – 162 | PET | 52 |
| 2 | FCA229 | CAAACTGACAAGCTTAGAGGGC | GCAGAAGTCCAATCTCAAAAGTC | 160 – 170 | PET | 52 |
| 3 | FCA24 | GGAAATTAGGAGCTCTGCCA | AAGATTTACCCAGTTGCCCC | 145 – 151 | PET | 52 |
| 2 | FCA310 | TTAATTGTATCCCAAGTGGTCA | TAATGCTGCAATGTAGGGCA | 121 – 137 | PET | 52 |
| 3 | FCA441† | ATCGGTAGGTAGGTAGATATAG | GCTTGCTTCAAAATTTTCAC | 153 – 183 | VIC | 56 |
| 3 | FCA453 | AATTCTGAGAACAAGCTGAGGG | ATCCTCTATGGCAGGACTTTG | 186 – 198 | NED | 56 |
| 3 | FCA678 | AGCAATCTCCAGAATGTGTGG | TCAAAAGATTAAAGCCTTCCAA | 226 – 234 | VIC | 56 |
| † Tetranucleotide repeat | | |  |  |  |  |

**Table A2.** Autocorrelated Kernel Density Estimates (95% AKDE) of leopard home range size within and between sexes for 142 known leopards within the Sabi Sand Game Reserve (SSGR) and Phinda-uMkhuze Complex (PMC), South Africa, 2002–2018. Parameter estimates are presented as the total number per category (N); mean home range size in square kilometers (x̅); standard errors (SE) and associated *P*-values are based on the *t*-statistic for independent variables (two-tailed), with Welch-correction for unequal variance, where 95% confidence intervals are presented (C.I.).

| **Category** | | **SSGR** | |  | **PMC** | |  | **Comparison** | | |
| --- | --- | --- | --- | --- | --- | --- | --- | --- | --- | --- |
|  |  | N | x̅ (±SE) |  | N | x̅ (±SE) |  | *t*_df_ | *P*-value | C.I. |
| Female | | 49 | 26.93 (2.37) |  | 31 | 31.54 (1.34) |  | 1.60_78_ | 0.113 | -10.36; 1.13 |
| Male | | 24 | 50.02 (5.43) |  | 38 | 50.32 (5.01) |  | 0.04_54_ | 0.968 | -15.12; 14.51 |
| All Individuals | | 73 | 34.50 (2.69) |  | 69 | 41.90 (3.05) |  | 1.81_136_ | 0.073˙ | -15.30; 0.61 |
| **SSGR** | Female / male |  |  |  |  |  |  | 3.90_32_ | <0.001*** | -35.16; -11.03 |
| **PMC** | Female / male |  |  |  |  |  |  | 3.57_44_ | <0.001*** | -29.41; -8.16 |
| Significance (˙*P* ≤ 0.10; **P* ≤ 0.05; ***P* ≤ 0.01; ****P* ≤ 0.001) | | | | | | |  |  |  |  |

**Table A3.** Pairwise distances (km) between home range centroids, as 95% Autocorrelated Kernel Density Estimates (AKDE), per dyad, confirmed kin-relationships, confirmed breeding pairs and all individuals for 142 known leopards within the Sabi Sand Game Reserve (SSGR) and Phinda-uMkhuze Complex (PMC), South Africa, 2002–2018. Parameter estimates are presented as the total number of pairs (N); mean distance in kilometers (x̅); standard errors (SE) and associated *P*-values are based on the *t*-statistic for independent variables (two-tailed), with Welch-correction for unequal variance, where 95% confidence intervals are presented (C.I.).

| **Category** | | **SSGR** | |  | **PMC** | |  | **Comparison** | | |
| --- | --- | --- | --- | --- | --- | --- | --- | --- | --- | --- |
|  |  | N | x̅ (±SE) |  | N | x̅ (±SE) |  | *t*_df_ | *P*-value | C.I. |
|  | All Individuals | 2628 | 13.40 (0.13) |  | 2346 | 13.23 (0.16) |  | 0.81_4719_ | 0.420 | -0.24; 0.58 |
| **Dyads** | Female – Female | 1176 | 12.90 (0.19) |  | 465 | 13.70 (0.35) |  | 1.94_775_ | 0.053˙ | -1.56; 0.00 |
|  | Female – Male | 1176 | 13.73 (1.99) |  | 1178 | 13.09 (0.22) |  | 2.15_2327_ | 0.032* | 0.06; 1.23 |
|  | Male – Male | 276 | 14.44 (0.43) |  | 703 | 13.28 (0.30) |  | 2.21_566_ | 0.028* | 0.13; 2.18 |
| **Kin-relationships** | Father – Daughter | 19 | 7.72 (2.15) |  | 17 | 5.61 (0.46) |  | 0.96_19_ | 0.348 | -2.49; 6.72 |
|  | Father – Son | 12 | 10.21 (1.58) |  | 23 | 10.86 (1.76) |  | 0.28_31_ | 0.784 | -5.47; 4.16 |
|  | Mother – Daughter | 27 | 4.61 (1.30) |  | 14 | 3.57 (0.68) |  | 0.71_36_ | 0.482 | -1.93; 4.02 |
|  | Mother – Son | 16 | 7.64 (1.64) |  | 18 | 3.54 (0.81) |  | 2.24_22_ | 0.036* | 0.30; 7.89 |
|  | Breeding Pair | 13 | 2.27 (0.51) |  | 12 | 9.70 (2.42) |  | 3.08_23_ | 0.005** | -12.43; -2.43 |
| **SSGR** | Father – Daughter / Father – Son | | | | | |  | 0.93_28_ | 0.359 | -7.95; 2.97 |
|  | Mother – Daughter / Mother – Son | | | | | |  | 1.45_32_ | 0.158 | -7.30; 1.24 |
| **PMC** | Father – Daughter / Father – Son | | | | | |  | 2.90_24_ | 0.008** | -9.00; -1.51 |
|  | Mother – Daughter / Mother – Son | | | | | |  | 0.03_29_ | 0.979 | -2.13; 2.19 |
| Significance (˙*P* ≤ 0.10; **P* ≤ 0.05; ***P* ≤ 0.01; ****P* ≤ 0.001) | | | | | | | | | | |

**Table A4.** Microsatellite diversity (22 loci and a sex-linked marker) of 142 known leopards within the Sabi Sand Game Reserve (SSGR) and Phinda-uMkhuze Complex (PMC), South Africa, 2002–2018. Parameter estimates are presented as the number of individuals successfully typed (N); number of alleles (*A*_n_); allelic richness (*A*_r_); number of private alleles (*A*_p_); observed heterozygosity (*H*_O_); expected heterozygosity (*H*_E_). In summary are shown: the mean (x̅); standard error (±SE) and total for each parameter and associated *P*-values are based on the ꭓ^2^ statistic of deviation from Hardy-Weinberg Equilibrium (HWE). FCA096 was removed from all further analyses due to poor amplification success (14–30% of individuals).

| **Locus** | **Sabi Sand Game Reserve (SSGR)** | | | | | | |  | **Phinda-uMkhuze Complex (PMC)** | | | | | | |
| --- | --- | --- | --- | --- | --- | --- | --- | --- | --- | --- | --- | --- | --- | --- | --- |
|  | N | *A*_n_ | *A*_r_ | *A*_p_ | *H*_O_ | *H*_E_ | *F_IS_* |  | N | *A*_n_ | *A*_r_ | *A*_p_ | *H*_O_ | *H*_E_ | *F_IS_* |
| FCA008 | 69 | 10 | 6.36 | 2 | 0.78* | 0.77 | -0.02 |  | 63 | 8 | 5.12 | 0 | 0.72 | 0.76 | 0.06 |
| FCA026 | 68 | 13 | 7.32 | 4 | 0.81 | 0.81 | -0.01 |  | 68 | 9 | 6.97 | 0 | 0.85 | 0.83 | -0.04 |
| FCA043 | 70 | 9 | 5.39 | 2 | 0.88 | 0.77 | -0.14 |  | 69 | 8 | 4.70 | 1 | 0.44* | 0.61 | 0.23 |
| FCA075 | 70 | 12 | 5.67 | 3 | 0.78* | 0.65 | -0.19 |  | 68 | 10 | 5.02 | 1 | 0.58* | 0.65 | 0.14 |
| FCA077 | 70 | 9 | 6.92 | 1 | 0.95 | 0.84 | -0.14 |  | 68 | 9 | 6.10 | 1 | 0.80 | 0.77 | -0.05 |
| FCA090 | 65 | 9 | 6.42 | 3 | 0.72* | 0.80 | 0.01 |  | 63 | 6 | 5.05 | 0 | 0.68* | 0.79 | 0.15 |
| FCA094 | 70 | 9 | 5.73 | 4 | 0.87* | 0.77 | -0.13 |  | 64 | 5 | 3.92 | 0 | 0.62 | 0.63 | 0.01 |
| ~~FCA096~~ | ~~11~~ | ~~13~~ | ~~9.00~~ | ~~11~~ | ~~0.54*~~ | ~~0.81~~ | ~~0.32~~ |  | ~~21~~ | ~~2~~ | ~~2.00~~ | ~~0~~ | ~~0.18*~~ | ~~0.17~~ | ~~-0.10~~ |
| FCA097 | 66 | 10 | 6.57 | 2 | 0.79 | 0.82 | 0.03 |  | 54 | 10 | 7.41 | 2 | 0.59* | 0.85 | 0.26 |
| FCA105 | 68 | 9 | 4.94 | 5 | 0.67* | 0.61 | -0.10 |  | 68 | 4 | 3.16 | 0 | 0.70 | 0.64 | -0.10 |
| FCA126 | 68 | 13 | 8.53 | 7 | 0.96 | 0.87 | -0.11 |  | 68 | 6 | 3.97 | 0 | 0.44* | 0.61 | 0.20 |
| FCA139 | 66 | 8 | 4.37 | 3 | 0.48 | 0.42 | -0.13 |  | 68 | 6 | 4.51 | 1 | 0.75 | 0.70 | -0.08 |
| FCA161 | 66 | 7 | 5.76 | 1 | 0.86 | 0.80 | -0.08 |  | 66 | 6 | 4.43 | 0 | 0.69 | 0.69 | -0.01 |
| FCA220 | 68 | 10 | 7.43 | 5 | 0.88 | 0.86 | -0.03 |  | 65 | 6 | 4.76 | 1 | 0.65* | 0.75 | 0.13 |
| FCA221 | 70 | 8 | 6.11 | 4 | 0.79 | 0.81 | 0.02 |  | 61 | 4 | 3.95 | 0 | 0.61* | 0.71 | 0.11 |
| FCA224 | 66 | 12 | 8.07 | 5 | 0.88 | 0.85 | -0.05 |  | 51 | 8 | 6.48 | 1 | 0.84* | 0.92 | 0.11 |
| FCA229 | 68 | 9 | 7.13 | 1 | 0.85 | 0.83 | -0.03 |  | 61 | 8 | 5.17 | 0 | 0.59* | 0.58 | -0.04 |
| FCA247 | 66 | 8 | 5.53 | 1 | 0.75* | 0.74 | -0.02 |  | 40 | 8 | 5.68 | 1 | 0.77* | 0.76 | -0.02 |
| FCA310 | 66 | 8 | 6.11 | 2 | 0.90 | 0.82 | -0.10 |  | 65 | 7 | 5.39 | 1 | 0.75* | 0.89 | 0.14 |
| FCA441 | 70 | 5 | 3.37 | 1 | 0.49 | 0.44 | -0.13 |  | 68 | 4 | 2.88 | 0 | 0.31 | 0.30 | -0.05 |
| FCA453 | 69 | 6 | 2.66 | 3 | 0.43 | 0.36 | -0.20 |  | 68 | 4 | 3.29 | 1 | 0.60 | 0.59 | -0.03 |
| FCA678 | 70 | 9 | 5.80 | 1 | 0.80* | 0.74 | -0.08 |  | 68 | 8 | 4.67 | 0 | 0.56* | 0.72 | 0.21 |
| **x̅** | **65.45** | **8.40** | **6.15** | **3.23** | **0.78** | **0.73** | **-0.08** |  | **61.59** | **6.64** | **4.76** | **0.50** | **0.65** | **0.70** | **0.06** |
| **SE** | **x2.62** | **0.46** | **0.32** | **0.51** | **0.03** | **0.04** | **0.02** |  | **x2.45** | **0.46** | **0.28** | **0.13** | **0.03** | **0.03** | **0.03** |
| **Total** | **-** | **206** | **-** | **71** | **-** | **-** | **-** |  | **-** | **146** | **-** | **-** | **-** | **-** | **-** |

**Table A5.** Parentage assignment success of 142 known leopards within the Sabi Sand Game Reserve (SSGR) and Phinda-uMkhuze Complex (PMC), South Africa, 2002–2018. Parameter estimates are presented as the percentage of individuals for which maternity was successfully assigned; the number of mothers this confirms from field-based observations; the percentage of individuals for with paternity was successfully assigned; the number of fathers this confirms from field-based observations; the percentage of individuals for which bi-parental assignment was not possible and the resolving power given the variation across these 22 loci within these two populations.

| **Category** | **SSGR** | **PMC** |
| --- | --- | --- |
| Maternity assigned | 62.90% | 47.80% |
| Field observed maternity confirmed | (30/30) | (25/31) |
| Paternity assigned | 54.30% | 64.20% |
| Field observed paternity confirmed | (20/20) | (16/28) |
| Assignment not possible (both parents) | 30.00% | 19.40% |
| Predicted resolving power of loci across sampled populations† | 99.00% | 96.00% |
| †Percentage of offspring for which CERVUS v 3.0.7 (Marshall et al. 1998) simulation predicts, based on loci sampled, assignment of parentage with 95% confidence. | | |
