# Supplementary figures and images for: Unsustainable anthropogenic mortality disrupts natal dispersal and promotes inbreeding in leopards
Source: Ecol Evol. 2020 Mar 18;10(8):3605–19. doi: 10.1002/ece3.6089 (PMC7160178; doi:10.1002/ece3.6089)

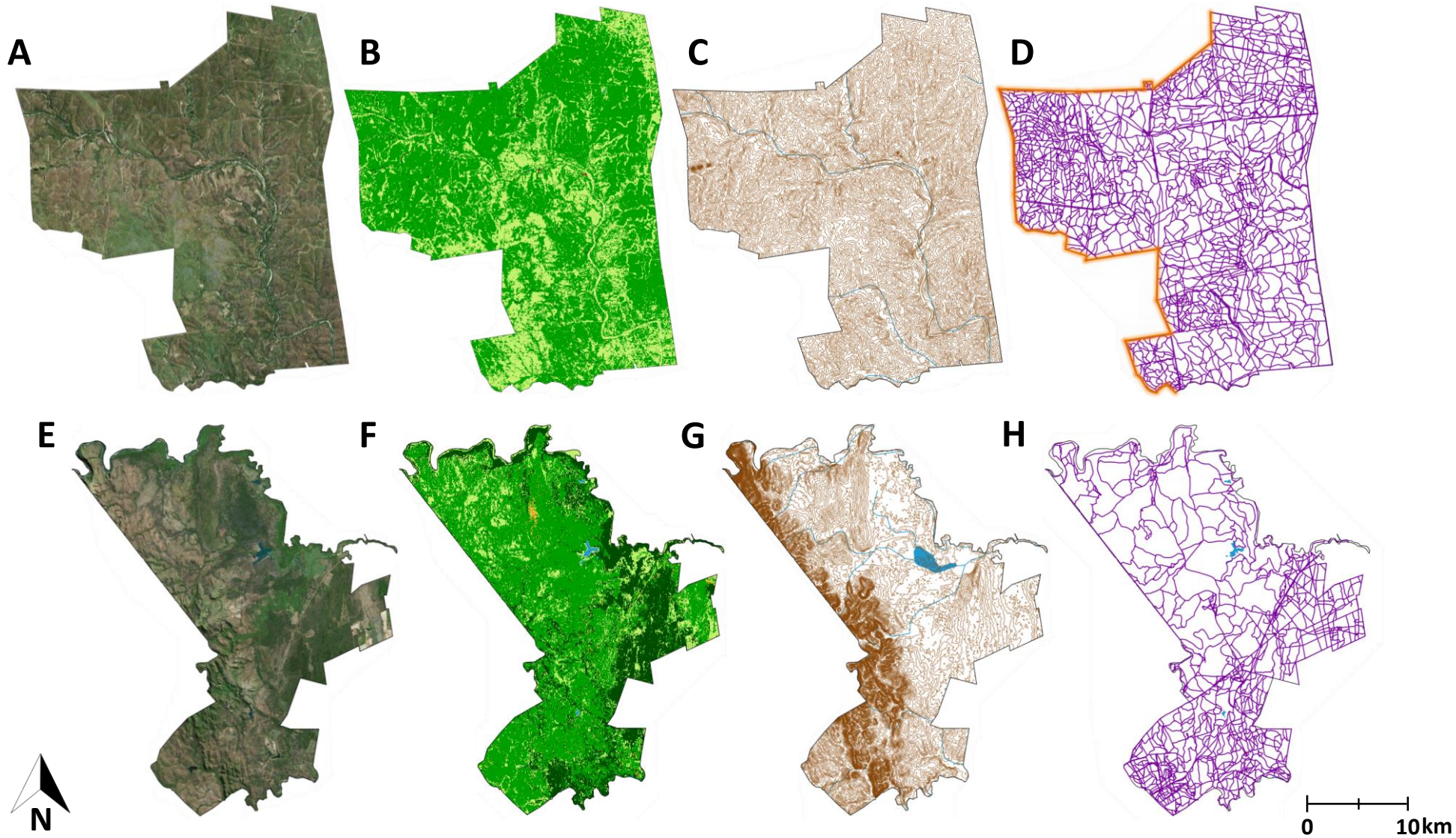

Supplement: Supplementary file 2 [file ECE3-10-3605-s002.pdf]
